# Pathogenic *DNM1L* Variant (1085G>A) Linked to Infantile Progressive Neurological Disorder: Evidence of Maternal Transmission by Germline Mosaicism and Influence of a Contemporary in cis Variant (1535T>C)

**DOI:** 10.3390/genes12091295

**Published:** 2021-08-24

**Authors:** Claudia Piccoli, Rosella Scrima, Annamaria D’Aprile, Massimiliano Chetta, Olga Cela, Consiglia Pacelli, Maria Ripoli, Giovanna D’Andrea, Maurizio Margaglione, Nenad Bukvic, Nazzareno Capitanio

**Affiliations:** 1 Department of Clinical and Experimental Medicine, University of Foggia, 71121 Foggia, Italy; claudia.piccoli@unifg.it (C.P.); rosella.scrima@unifg.it (R.S.); olga.cela@unifg.it (O.C.); consiglia.pacelli@unifg.it (C.P.); giovanna.dandrea@unifg.it (G.D.); maurizio.margaglione@unifg.it (M.M.); 2 Cytogenetic Unit, Azienda Ospedaliera Universitaria, Ospedali Riuniti, 71121 Foggia, Italy; annamariadaprile@libero.it; 3 U.O.C. Genetica Medica e di Laboratorio, Ospedale Antonio Cardarelli, 80131 Napoli, Italy; m_ax@libero.it; 4 Production Unit of Advanced Therapies (UPTA), Institute for Stem-Cell Biology, Regenerative Medicine and Innovative Therapies (ISBReMIT), I.R.C.C.S. Casa Sollievo della Sofferenza Hospital, 71013 San Giovanni Rotondo, FG, Italy; m.ripoli@operapadrepio.it; 5 Medical Genetic Unit, Azienda Ospedaliero Universitaria Consorziale Policlinico di Bari, 70124 Bari, Italy

**Keywords:** DNM1L, Drp1, mitochondrial fission, encephalopathy, genetic mosaicism

## Abstract

Mitochondria are dynamic organelles undergoing continuous fusion and fission with Drp1, encoded by the *DNM1L* gene, required for mitochondrial fragmentation. *DNM1L* dominant pathogenic variants lead to progressive neurological disorders with early exitus. Herein we report on the case of a boy affected by epileptic encephalopathy carrying two heterozygous variants (*in cis*) of the *DNM1L* gene: a pathogenic variant (PV) c.1085G>A (p.Gly362Asp) accompanied with a variant of unknown significance (VUS) c.1535T>C (p.Ile512Thr). Amplicon sequencing of the mother’s DNA revealed the presence of the PV and VUS in 5% of cells, with the remaining cells presenting only VUS. Functional investigations performed on the patient and his mother’s cells unveiled altered mitochondrial respiratory chain activities, network architecture and Ca^2+^ homeostasis as compared with healthy unrelated subjects’ samples. Modelling Drp1 harbouring the two variants, separately or in combination, resulted in structural changes as compared with Wt protein. Considering the clinical history of the mother, PV transmission by a maternal germline mosaicism mechanism is proposed. Altered Drp1 function leads to changes in the mitochondrial structure and bioenergetics as well as in Ca^2+^ homeostasis. The novel VUS might be a modifier that synergistically worsens the phenotype when associated with the PV.

## 1. Introduction

The morphology of the cellular mitochondrial compartment is dictated by a dynamic equilibrium between the fusion and fission of the organelle. The prevalence of fusion leads to highly interconnected tubular mitochondria, whereas the prevalence of fission leads to more fragmented isolated mitochondria [1,2]. In recent decades, the molecular details of fusion/fission have been progressively identified, although some mechanistic features remain to be clarified. The morphological status of mitochondria is reciprocally linked to several fundamental cellular processes [3,4], therefore it is not surprising that defective fusion/fission impacting on specific cell homeostasis has been linked to the development of diseases [5]. Different genetic variants have been described in several coding genes involved in mitochondrial dynamics and most of them are associated with congenital encephalopathies [5,6,7]. *DNM1L* (DYNAMIN 1 LIKE; OMIM 603850) is a gene coding for the dynamin related protein 1 (Drp1), an essential component of the mitochondrial fission machinery, associated with lethal encephalopathy (OMIM 614388). To date, 15 different variants of *DNM1L* have been described that led to the loss of function of Drp1; they may be inherited in a recessive or dominant manner, or arise de novo. For example, the variants c.1184C-A (p. Ala395Asp) [8], c.1085G-A (p.Gly362Asp) [9], c.1084G-A (p.Gly362Ser) [10] in the *DNM1L* gene acted in a dominant-negative manner. However, autosomal recessive encephalopathy with compound heterozygous mutations in the *DNM1L* gene have also been reported, i.e., c.261dup; p.Trp88MetfsTer9, and c.385_386del; p.Glu129LysfsTer6 [11], just as reported in [12], c.346_347delGA (p.Glu116LysfsTer6) and a c.106A-G (p.Ser36Gly) (see also https://www.omim.org/entry/603850, accessed on 29 January 2021). The clinical phenotypes of patients harbouring variants of *DNM1L* are similar, with poor prognosis leading to exitus within early childhood.

**Figure 1:**
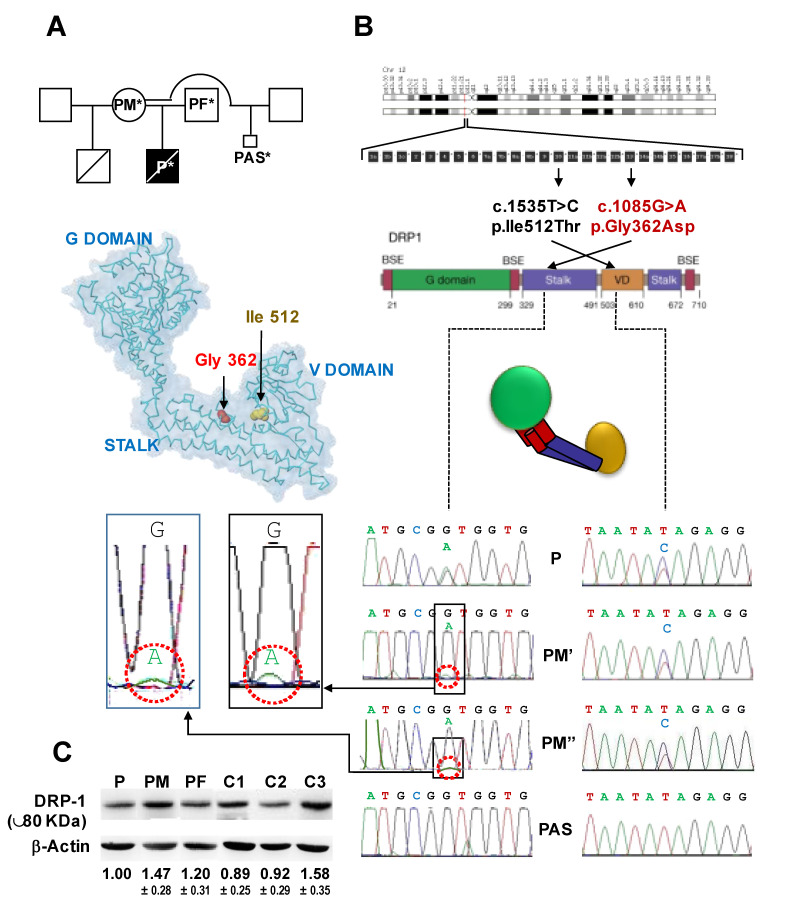
Mutational analysis of *DNM1L*. (**A**) Pedigree of the patient family. P*, patient; PM*, patient’s mother; PF*, patient’s father; PAS*, patient’s aborted sibling. Subjects whose biological samples were made available for this study are shown; the smaller square refers to a third conceived by PM deliberately aborted. (**B**) From the top down: positioning of the DNM1L gene on chromosome 12; gene structure with the two indicated nucleotide variants object of this study; protein domains in the Drp1 primary structure and their schematic assembly in the protein folded structure along with the backbone structure of Dpr1 (left side) modelled using the coordinates of the PDB ID: 3W6O and shown with the indicated domains and location of the variant residues (coloured space-filled rendering); electropherograms showing the DNM1L variants of PBMC of the patient (P) and patient’s mother (PM’), in the saliva of the patient’s mother (PM’’) and in the chorionic villi fibroblast of the patient’s aborted sibling (PAS); enlargements of the PM’ and PM’’ electropherograms are shown at the side. (**C**) Immunoblotting of PBMC protein extract against anti-Drp1 and β-actin: P, PM as in panel (**B**); PF, patient’s father; C1, C2, C3 unrelated healthy controls; the figures below each lane are the averaged densitometric values (±SEM) normalized to P from three independent assays.

In this study we reassessed the case of a boy affected by epileptic encephalopathy already described in the literature by an independent group [13] and harbouring a known pathogenic variant of *DNM1L* and a novel variant of uncertain significance (VUS) on the same allele (*in cis*). Integrating DNA sequence analyses on blood samples of the patient and his mother and on cultured fibroblasts obtained from placenta (chorionic villus) of the mother’s last interrupted pregnancy (see pedigree in Figure 1A), herein the hypothesis of a matrilineal transmission of the *DNM1L* variant by germline mosaicism has been discussed. Moreover, assessment of mitochondria-related functions and in silico modelling strongly suggested that coexistence of the VUS in the *DNM1L* gene might be considered an additive condition, worsening the phenotype associated with the pathogenic variant.

## 2. Materials and Methods

### 2.1. Samples

Cell samples were obtained from patients’ (a 10-year-old boy (P) and his mother (PM)) and healthy volunteers’ peripheral venous blood. Peripheral blood mononucleate cells (PBMCs) were isolated by standard procedure in phosphate-buffered saline (PBS), counted and immediately assayed or frozen at −80 °C. PM buccal mucosa cells were collected by swab under sterile conditions and DNA isolated by QIAmp DNA Mini Kit (Qiagen, Hilden, Germany) according to the manufacturer’s instructions. Fibroblasts from chorionic villus of abortive sample were isolated and cultured as previously described [14]. The patient’s mother provided written informed consent for the study and publication of this article in agreement with the Declaration of Helsinki.

### 2.2. Measurement of Respiratory Activity

Freshly isolated PBMCs were resuspended in 10 mM KH_2_PO_4_, 27 mM KCl, 1 mM MgCl_2_, 40 mM Hepes, 0.5 mM EGTA, pH 7.1 at 4 × 10^6^ cells/2 mL and immediately assayed for O_2_ consumption by high resolution oxymetry (Oxygraph-2k, Oroboros Instruments) at 37 °C under continuous stirring; the oxygen consumption rate (OCR) was attained as a negative derivative of the O_2_ content changes over time. See Supplemental Materials for further details.

### 2.3. Measurement of Enzymatic Activities

Pelleted PBMCs were resuspended in 0.32 M sucrose, 40 mM KCl, 20 mM Tris-HCl, 2 mM EGTA, pH 7.2, frozen at −80 °C for 10 min, thawed at room temperature and subjected to six cycles of 10 sec sonication (>20 kHz). The specific enzymatic activities of the NADH: ubiquinone oxidoreductase (complex I), the ubiquinol: cytochrome c oxidoreductase (complex III), the cytochrome c oxidase (complex IV) and citrate synthase were assayed spectrophotometrically in 10 mM Tris-HCl, 1 mg/mL BSA, pH 8.0 as described [15]. The activities were normalized to the initial cell number or to cellular protein content.

### 2.4. Live Cell Imaging of ROS, Mitochondrial Membrane Potential, and Ca^2+^

PBMCs were incubated for 20 min at 37 °C with the following probes (Molecular Probes): 2 μM tetramethylrhodamine, ethyl ester (TMRE) to monitor mitochondrial membrane potential (mtΔΨ); 10 μM 2′,7′-dichlorofluorescin diacetate (DCF-DA) for detection of reactive oxidant species; 5 μM X-Rhod-1 AM for mitochondrial Ca^2+^. Stained cells were examined by a Nikon TE 2000 microscope (images collected using a 60X objective (1.4 NA)) coupled with a Radiance 2100 dual-laser confocal laser scanning microscopy system (Biorad) (see Supplemental Materials for further details).

### 2.5. Reverse Transcription-Polymerase Chain Reaction

For real-time reverse transcriptase (RT)-PCR, total cellular RNA was isolated, reverse transcribed by random hexamer primers and amplified as detailed in Supplemental Materials. mtDNA copy number quantification is also described in Supplemental Materials.

### 2.6. Western Blotting Analysis

A total of 40 μg of proteins from each cell lysate were subjected to SDS-PAGE, transferred to a polyvinylidene difluoride membrane and probed with primary and secondary antibodies as detailed in the Supplemental Materials.

### 2.7. DNM1L Gene Sequencing

The variants of *DNM1L* gene (NM_012062.3) were analysed by polymerase chain reaction (PCR)-generated amplicons of DNA extracted either from PBMCs, PM’s saliva or chorionic fibroblasts [16] and processed by an automated DNA sequencing analyser as specified in Supplemental Materials.

### 2.8. Structural Modelling of the Wt and Mutated Drp1

The monomer, homodimer and heterodimer structures of Wt and of Gly362Asp and/or Ile512Thr Drp1 variants were modelled, using as a template the Drp1 crystal structure (PDB ID: 3W6O), by Phyre2, GalaxyHomomer and GRAMM-X software, respectively; see Supplemental Materials for further details.

### 2.9. Statistical Analysis

All the experiments were carried out at least in triplicate. Data are shown as mean ± standard error mean (SEM) and compared by unpaired Student’s *t*-test or one-way ANOVA, followed by Bonferroni post hoc test; a * *p*-Value < 0.05 was accepted as statistically significant. All analyses were performed using Graph Pad Prism (Graph Pad software).

## 3. Results

We report on a 42 year old woman (patient mother—PM) who passed through our Genetic Counselling due to positive anamnestic data regarding the presence of an intellectual disability, transient ischemic episodes and myoclonic seizures in two sons generated by different fathers. The first son died at the age of three years (DNA samples not available), the second son (patient—P) died at 11 years (DNA samples available). A third pregnancy, by a still different father, was voluntarily terminated because of the unclear familiar clinical history (DNA from abortive samples available) (Figure 1A). Multiple clinical, genetic and metabolic investigations were undertaken in P reporting negative results. Verrigni et al. [13] by next generation sequencing (NGS) analysis on a panel of 224 genes associated with mitochondrial diseases identified two heterozygous variants of the *DNM1L* gene in P (Pt.4 in [6]): c.1085G>A (p.Gly362Asp)—PS1,PS3,PS4,PP3,PP4 and c.1535T>C (p.Ile512Thr)—BS2,BP2,BP4. The first variant (c.1085G>A) was already described as pathogenic and associated with an autosomal dominant encephalopathy [9] whereas the second (c.1535T>C), classified as VUS, has never been reported. When parents segregation (Sanger sequencing) was performed, of the two *DNM1L* variants only the VUS (c.1535T>C; p.Ile512Thr) was revealed in the mother’s DNA but neither in the father’s DNA.

The occurrence in the PM’s firstborn of epileptic encephalopathy led us to suspect matrilinear transmission of other variants of the mtDNA. X-linked transmission was excluded based on performed genetic testing. However, sequencing of mtDNA in P resulted in negative results. A number of already reported nucleotide base changes were observed both in coding and non-coding regions of the mtDNA. In the former case, synonymous base changes or variations leading to non-pathogenic aminoacid substitutions were detected (Table S1).

Using an NGS approach, Verrigni et al. revealed the c.1085G>A (p.Gly362Asp) variant in the PM blood sample DNA at very low levels (about 5%) and, by subcloning PCR products from P cDNA, the occurrence of both the DNM1L variants (in cis) on the same allele [13]. We verified and confirmed on selected *DNM1L* amplicons what had been reported and extended the sequence analysis on DNA extracted from the PM buccal swap and on the abortive tissue (chorionic villus fibroblasts) of the child conceived by PM with a third father (Figure 1B). The result attained sequencing saliva DNA from PM substantially confirmed what had been found in PM PBMCs (i.e., about 5% of the c.1085G>A variant while the heterozygotic c.1535T>C VUS was clearly detected). In the case of the abortive sample, no variants were observed in the *DNM1L* gene. As already performed genetic testing excluded X-linked transmission, the obtained results would indicate the occurrence of maternal somatic and germline mosaicism. It is noteworthy that Dpr1 expression was not significantly affected in blood samples of P as compared with PM, his father (PF) and three unrelated healthy subjects (Cs) (Figure 1C).

**Figure 2:**
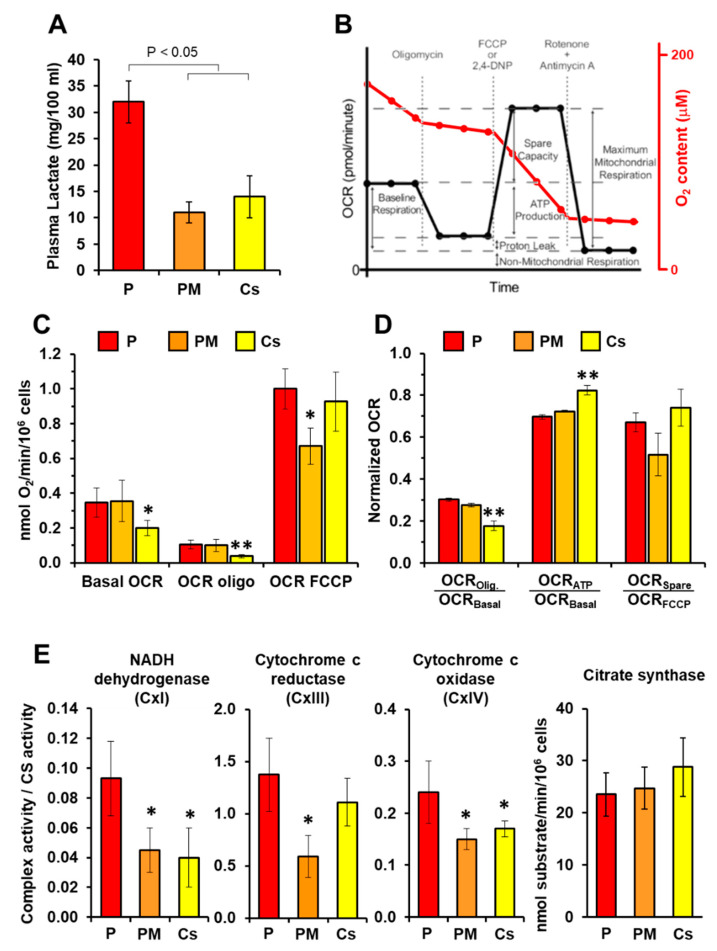
Analysis of the metabolic phenotype in PBMCs. (**A**) Plasma lactate content in patient (P), patient’s mother (PM) and the averaged value of four independent healthy controls (Cs). (**B**) Scheme showing the protocol utilized to assay mitochondrial respiratory activity in intact PBMCs cells by high resolution oxymetry; red trace, changes of oxygen content in the assay buffer under baseline and following sequential addition of oligomycin, FCCP and rotenone plus antimycin A; black trace, corresponding oxygen consumption rates (OCRs). (**C**) OCR/cell number under basal condition, in the presence of oligomycin (proton leak) and in the presence of FCCP (maximum respiration); the reported value are means ± SEM of three independent replicates and are corrected for the rotenone plus antimycin A-insensitive OCR; * *P* < 0.05 vs. P. (**D**) Normalized OCRs calculated from the data reported in panel (**C**); ** *P* < 0.01. (**E**) Activities of the respiratory chain complexes I, III and IV and citrate synthase in PBMC cell lysates; the activities were assessed by spectrophotometric assay and the reported values are means ± SEM of three independent replicates; * *P* < 0.05 vs. P. See Materials and Methods and Results for further details.

Blood samples from P and PM were available and completely independent analyses in parallel to and unaware of the Verrigni study [13] were performed. Plasma lactate content, under normal conditions, was almost three-fold higher in P as compared with PM and three unrelated healthy subjects (Figure 2A). Next, we carried out a systematic analysis of mitochondria-related functions. PBMCs were isolated and assayed by high resolution oxymetry (Figure 2B). As shown in Figure 2C the basal resting oxygen consumption rate (OCR) was significantly higher both in P and PM with respect to unrelated controls (averaged activity of three healthy subjects). Addition of the H^+^-ATP synthase inhibitor oligomycin depressed respiration, as expected, but more significantly in the control cells. Further addition of the protonophore uncoupler FCCP resulted in the maximal respiration that was comparatively lower in PM PBMCs. Normalization to the basal or maximal OCR, to compensate for inter-individual variability, confirmed a relatively higher proton leak and a lower ATP-linked OCR in P and PM (Figure 2D). The reported activities were corrected for the residual OCR after the combined addition of antimycin A and rotenone and are, therefore, ascribable to the mitochondrial respiratory chain. Measurement of the enzymatic activity of complexes I (NADH dehydrogenase), III (cytochrome c reductase), and IV (cytochrome c oxidase) normalized to that of the citrate synthase, as an indirect measure of the mitochondrial mass, resulted in higher values in all the complexes of P as compared with PM and controls (Figure 2E); the differences reached the larger statistical significance for complexes I and IV. Conversely, the citrate synthase activity did not show significant differences.

**Figure 3:**
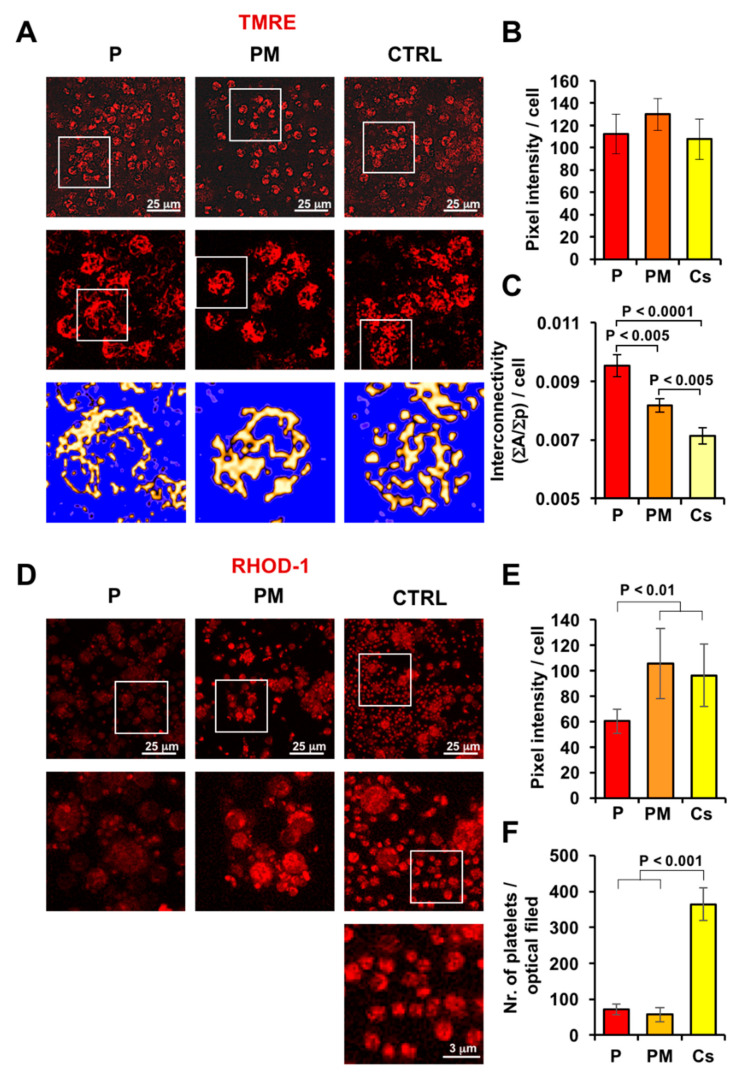
Morpho-functional analysis of the mitochondrial compartment. (**A**) Confocal microscopy imaging of mtΔΨ assessed by TMRE in PBMCs from patient (P), patient’s mother (PM) and a representative healthy subject (CTRL); the tripartite images show representative images and progressive digital magnifications of the squared details; images at the bottom are rendered as false colour. (**B**) Quantitative analysis of the TMRE fluorescence/cell. (**C**) Morphometric evaluation of the TMRE signal/cell with indicated statistical analysis. (**D**) Confocal microscopy imaging of mitochondrial Ca^2+^ in PBMCs assessed by Rhod-1; the tripartite images show representative images and digital magnifications of the squared details, and in the CTRL sample a further magnification is reported to highlight the platelets. (**E**) Histogram showing quantification of the Rhod-1 fluorescence intensity/cell. (**F**) Histogram showing quantification of the platelet count/optical field. In (**B**,**C**,**E**,**F**) at least five different optical field/samples, each containing 40–50 cells, were examined and the values averaged ± SD from three independent biological preparations with statistical indication when significant.

Next, we complemented the mitochondrial functional analysis with confocal microscopy imaging of the mitochondrial membrane potential (mtΔΨ) using the mito-tropic fluorescent probe TMRE (Figure 3A) in viable PBMCs. The results attained clearly showed an intracellular compartmentalization of the fluorescent signal highlighting the mitochondrial network. However, no significant difference in the averaged fluorescence intensity/cell was observed between P and PM and control samples (Figure 3B), indicating a similar ability to maintain the respiration-driven mtΔΨ.

However, a closer analysis of the images showed a different morphology of the mitochondrial compartment. In particular, mitochondria appeared more aggregated in P and PM and perinuclearly located, whereas they were more fragmented in all controls tested (Figure 3A). Morphometric analysis of the imaged mitochondria per cell confirmed a significantly higher interconnectivity index in P as compared with control samples with that of PM, displaying an intermediate value (Figure 3C); higher scores of this morphometric parameter signify that mitochondria have more physical connections, while lower scores signify that mitochondria are more fragmented.

Of note, in P-PBMCs the mtDNA copy number/cell was significantly lower (almost halved) than that of PM and controls (Figure S1A). Accordingly, the expression of PGC1-α, a master transcription factor controlling mitochondrial biogenesis, was significantly down-regulated in P as compared with PM and controls (Figure S1B).

Furthermore, we evaluated by the probe DCF the cellular redox state, as mitochondria are a major source of reactive oxygen species with their production frequently increased under conditions of mitochondrial respiratory chain dysfunction [17]. Additionally, in this case no significant change in the constitutive level of reactive species was observed among the different PBMC samples tested (Figure S2).

In addition to control ATP production by the oxidative phosphorylation, mitochondria are involved in the cellular Ca^2+^ homeostasis and signalling [18]. To investigate this function, we used the fluorescent probe Rhod-1, specifically detecting intra-mitochondrial Ca^2+^. As shown in Figure 3D,E the Rhod-1-related signal/cell resulted in significantly lower P-PBMCs as compared with both PM and control samples indicating a reduced ability of P mitochondria to uptake/retain Ca^2+^ under steady state condition. Notably, a closer inspection of the optical fields of Rhod-1-loaded PBMC in controls unveiled the presence of extracellular signals in smaller organelles, likely attributable to platelet mitochondria. The presence of Rhod-1-loaded platelet mitochondria was much more scarce in P and PM and had a more aggregated appearance therein (Figure 3D,F).

Multiple sequence alignment of Drp1 showed that the G362 residue was totally conserved among all eukaryotes, whereas the I512 residue was conserved only in chordate (Figure S3). Mapping of the two residues in the crystal structure of Drp1 localizes G362 in a portion of the protein shown to be involved in binding of mitochondrial receptors as well as in the inter-monomer interactions governing the dimer/oligomer geometry [4]. The I512 residue was located in an unstructured mobile domain of the protein (illustrated in Figure 1B as a modelled structure) that was shown to inhibit the assembly of the monomers by capping the interface where G362 is located [19]. Substitution of I512 with T512 did not apparently generate a putative threonine-phosphorylation site (assessed by the NetPhos 3.1 server—http://www.cbs.dtu.dk/services/NetPhos/, accessed on 29 January 2021).

**Figure 4:**
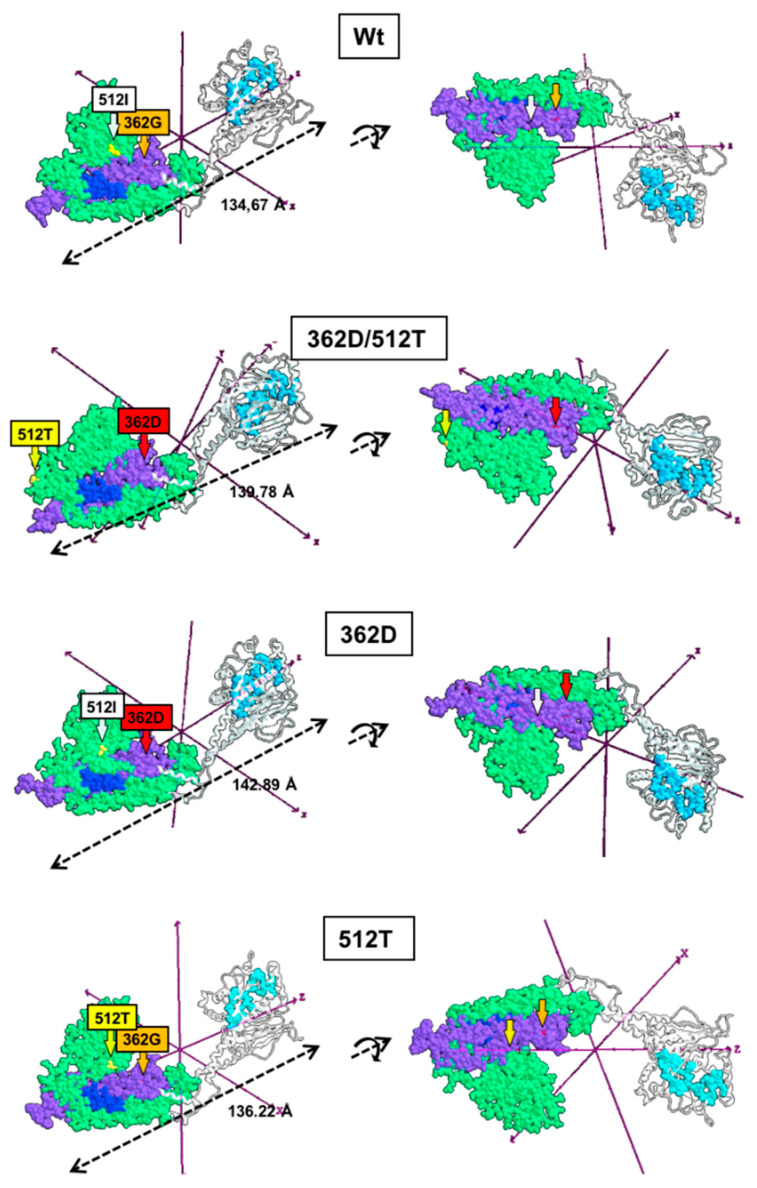
Modelling of the monomeric structure of Wt Drp1 and 362D, 512T variants. The mixed rendered structures were modelled using the coordinates of the PDB ID: 3W6O; two projections rotated by 180° on the X axis are shown. Light blue, residues contributing to the GTP binding site; violet, middle domain (aa 344–489); light green, GSK3β-interaction domain (aa 448–685); blue, homodimerization domain (aa 654–668). The tagged Wt residues 362G and 512I are arrowed in orange and white, respectively; the variants 362D and 512T are arrowed in red and yellow, respectively.

The structural effects of the variants 362Asp and 512Thr, separately and in combination, were modelled using the 3D-structure of the Wt-Dpr1 as a starting template (PDB ID: 3W6O) (Figure 4). Comparison of the modelled proteins unveiled variations in the structure of the GSK3β-interaction domain (aa 448–685), which could affect the GTPase activity; indeed, interference with the GSK3β-mediated phosphorylation of Ser693 inhibits mitochondria fragmentation [20]. Most notably, the larger change in the GSK3β-binding domain was observed in the modelled structure of Drp1 harbouring both the 362D and the 512T variants with a complete delocalization of the 512Thr residue. The large dislocation of 512T was not observed in the modelled protein harbouring the single variants, pointing to a combined effect. Variations in the length of the protein variants were also observed (Figure 4).

**Figure 5:**
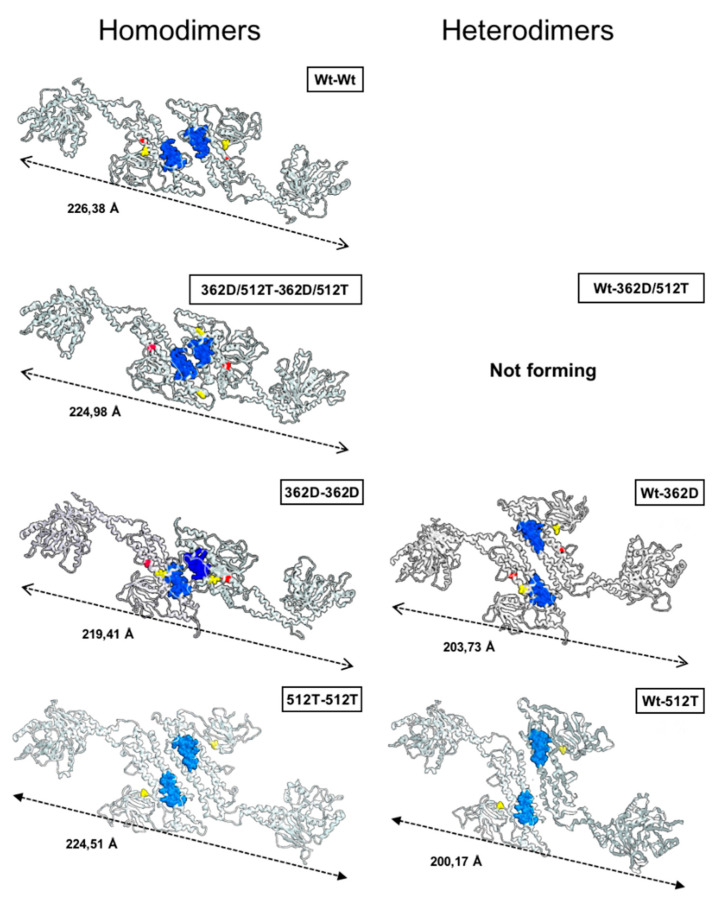
Modelling of the dimeric structure of Wt Drp1 and 362D, 512T variants. The mixed rendered dimeric structures were modelled starting from the monomeric coordinates of the PDB ID: 3W6O. The modelled dimers were constructed using identical monomers (homodimers) or a combination of a Wt monomer and a mutant monomer carrying one or the other of the single variants or a combination of both (heterodimers). The domain essential for dimerization is shown in blue (aa 654–668); residues at position 362 (G or D) and 512 (I or T) are show in red and yellow, respectively.

Afterward, formation of homodimeric (wt–wt, 362Asp/512Thr-362Asp/512Thr, 362Asp-362Asp and 512Thr-512Thr) and heterodimeric (wt-362Asp/512Thr, wt-362Asp and wt-512Thr) complexes was evaluated (Figure 5). Modelling of Drp1 homodimers resulted in different dimer lengths, likely caused by changes in the intermonomer surface interaction (aa 654–668) necessary for dimerization [21]. The length changes in the homodimers 362Asp/512Thr-362Asp/512Thr (occurring in P) and 512Thr-512Thr (occurring in PM) as compared with the Wt–Wt dimer, could affect the stability of the Drp1 spiral hetero-oligomeric structures. The proficiency of Wt Drp1 protein to form heterodimer with that harbouring the two variants, singularly or combined, resulted completely or largely hindered (i.e., 0% for Wt-362Asp/512Thr, 4% for Wt-362Asp, 2% for Wt-512Thr) with shortening of the heterodimer length when formed (Figure 5). No substantial changes were observed in the conformation of the GTPase domain.

## 4. Discussion

In addition to the 37 mtDNA encoded genes, about 2000 mitochondria-related nuclear genes exist and many of their variants have been identified and linked to diseases indirectly linked to bioenergetic failure [22,23] with the highest energy-demanding tissues/organs, such as the brain, being more affected, particularly during development [24,25].

The mitochondrial compartment is shaped by a continuous flux of organelle fusion and fission, orchestrated by interactive signalling pathways adapting the cell to internal and external cues. In particular, proper mitochondrial fission has been linked to cell division, organelle biogenesis, quality control via mitophagy or apoptosis, oxidative metabolism, ageing and senescence, immunity, inter-organelle interaction/signalling, and differentiation [1,4]. The *DNM1L-*encoded GTPase Drp1 is the major executioner of the mitochondrial fission; it exists as dimer/tetramer in the cytosol and once recruited to the outer mitochondrial membrane forms helical oligomers that induce membrane constriction and severing with the mechano-chemical driving force provided by GTP hydrolysis.

In this study, we re-appraised the reported case of a boy affected by epileptic encephalopathy carrying heterozygotic variants of the *DNM1L* gene [13] and provide additional evidence about (a) the modality of hereditary transmission, (b) the impact of the variants on mitochondria-related functions, and (c) predictive models of the protein structure.

**Figure 6:**
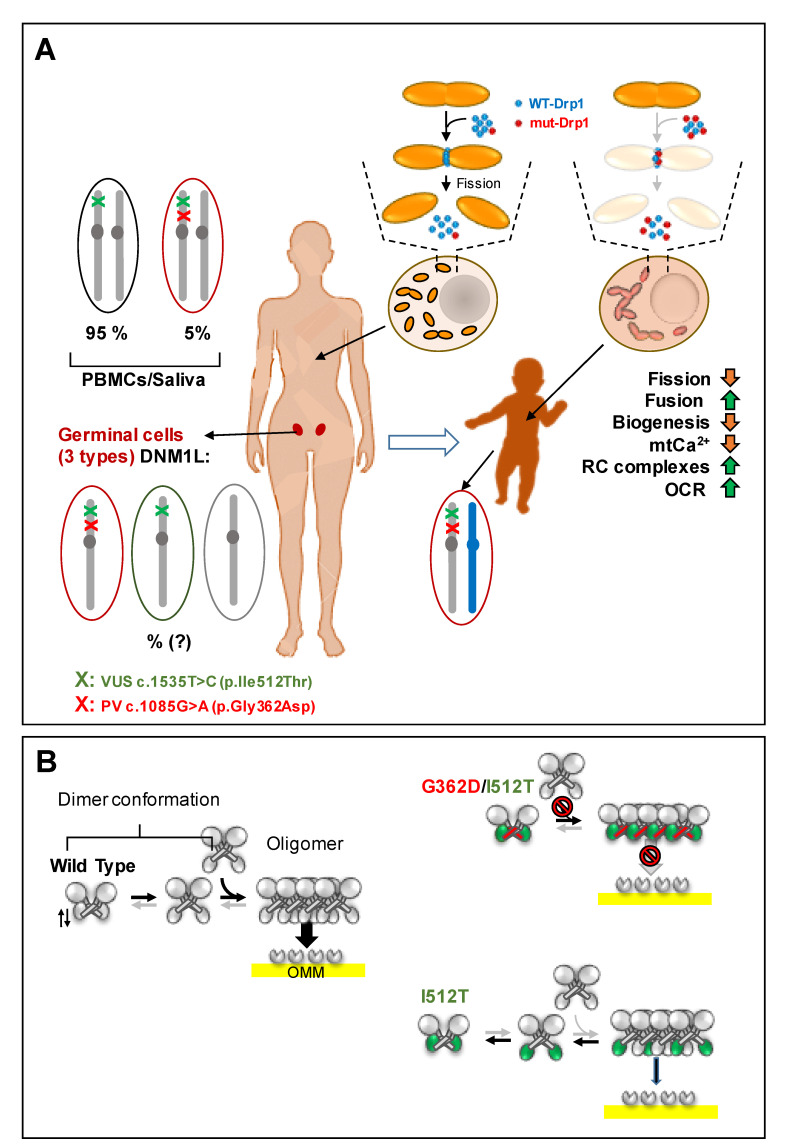
Schematic representation of the proposed pathogenic mechanism linked to the Drp1 variants that are the object of the present study. (**A**) Model of the mosaicism-based hereditary (AD—autosomic dominant) transmission of the pathogenic variant (PV) and VUS *DNM1L in cis* variants; mosaicism refers to the presence in an individual of genotipycally distint cells derived from a single zygote (post zygotic event). (**B**) Model of the impact of the Drp1 variants on the dimer conformations equilibrium and oligomeric assembly and binding to the outer mitochondrial membrane (OMM). See Discussion for further explanation.

Sequence analysis carried out on PBMC DNA amplicons from P and PM confirmed the presence in *DNM1L* of the two heterozygotic variants c.1085G>A (p.Gly362Asp) and c.1535T>C (p.Ile512Thr). The first variant (PV) was present in all the patient’s blood cells (100%) and only in about 5% of the mother’s PBMCs and saliva; the second (VUS) was present in both P and PM PBMCs and in PM saliva, confirming what was reported for the dermal fibroblast [13]. An analysis of an abortive sample of a third conceived of the mother, with a different father, showed the presence of Wt *DNM1L*. Considering the medical history of the apparently unaffected mother who had a first son—with a yet different father—exhibiting an encephalopathic syndrome as in P, it is reasonable to hypothesize a condition of germline mosaicism. In this scenario, the ensuing haploid oocytes should result in three subpopulations: one with both the c.1085G>A p.Gly362Asp and c.1535T>C p.Ile512Thr variants of *DNML1*; one with only the c.1535T>C p.Ile512Thr variant; and one with a Wt *DNML1*. The percentage of the three oocyte subsets has to be considered unknown/unpredictable (Figure 6A).

Mosaicism can complicate clinical diagnosis and genetic counselling. Regardless of mosaicism type in parents, whether germline or somatic, the risk of recurrence of another conceived with the disease exists and has to be taken into account [26]. Probabilistic modelling of gametogenesis predicts that mutations in parental blood increases recurrence risk substantially more than parental mutations confined to the germline [26]. Mosaic phenotypes may have incomplete or even unnoticed syndromic features and mutational load in the material tested does not necessarily correlate with the severity of the disease [27]. Moreover, somatic mutations can possibly affect the epigenetic patterns and levels of gene expression contributing to the pathogenesis of certain disorders [28,29]. Taking all of this into consideration, along with what is here reported, long time follow-up of subjects such as the patient’s mother is recommended.

In the studies reporting one or the other of the fifteen Drp1 pathogenic variants so far identified, only in a few has their impact on the mitochondrial respiratory activity been assessed, mainly in cultured patients’ fibroblasts or muscle homogenate, with contrasting results. To the best of our knowledge, this is the first study where measurement of the mitochondrial respiratory activity in intact primary cells of a Drp1-mutated patient is reported. Surprisingly, the mitochondria-related OCR in patient PBMCs resulted in almost twice that measured in control cells. Accordingly, the enzymatic activities of the respiratory chain complexes I, III and IV appeared to be significantly higher in patient PBMCs.

Mitochondrial morphology might influence the respiratory chain activity [30,31]. This appears to be linked to the remodelling of the inner mitochondrial membrane cristae where the respiratory chain complexes are mainly localized [2]. A shift toward a more fused mitochondria appears to promote nutrient utilization and to enhance mitochondrial oxidative phosphorylation; on the contrary, fragmented mitochondria are associated with reduced respiratory activity. However, it must be considered that mitochondrial fission is also a precondition for the organelle quality control whereby damaged mitochondria are segregated from the network and booted to mitophagic degradation [32]. While in non-dividing cells, such as neurons or myocytes, reduced fission would result in progressive accumulation of dysfunctional mitochondria [33], other cell types with a relatively short lifespan, such as PBMCs, might be less affected. Therefore, in the context of reduced Drp1 activity, such as in the patient’s PBMCs, the hyperfused-mitochondria, observed in the present study, would elicit higher respiratory competence, overcoming the presence of non-fully functional units in the organelle network. However, the relatively high level of plasma lactate in the patient would indicate the occurrence of more generalized systemic metabolic alterations.

In addition to providing energy, mitochondria contribute to regulating Ca^2+^ homeostasis and an interplay between mitochondrial dynamics and Ca^2+^ signalling is emerging in the playground of cell death and survival modulation [34]. In this report, we show that patient’s PBMCs displayed a much lower intra-mitochondrial basal Ca^2+^ content. A major source of mitochondrial Ca^2+^ is provided by the ER store via specialized contact sites (known as mitochondrial-associated membranes, MAMs) [35]. Since the site of fission is primed by the wrapping of ER around mitochondrial tubules [36], it is possible that dysfunction of the mitochondrial division machinery might indirectly affect the Ca^2+^-related cross-talk between the two compartments.

Incidentally, confocal microscopy imaging of the PBMCs, utilizing the mitochondrial Ca^2+^ probe, unveiled fewer platelets in P and PM samples. This is not surprising since proper mitochondrial fission is expected during maturation of megakaryocytes. Indeed, it has been reported that transgenic mice conditionally Drp1-knocked out in megakaryocytes resulted in reduced platelet count and altered functions [37].

The fission of mitochondria appears to be coordinated with mtDNA replication and the Drp1-mediated constriction sites co-localize at sites adjacent to replicating mitochondrial nucleoids [38]. Dysregulation of mitochondrial fission affects mtDNA replication, resulting in loss of mtDNA integrity and copy number and in its altered distribution in the mitochondrial network [39]. Consistently, we found a ≈50% reduction in the mtDNA copy number in the patient’s PBMCs in agreement with the reported higher frequency of *petite* clones in yeast strains harbouring the Drp1-homologous Dnm1 mutated in amino acids corresponding to the human G362D and/or I512T variants [13]. Accordingly, the expression of PGC1-α, the master positive regulator of mitochondrial biogenesis [40], also resulted in reduced PBMCs.

Crystallographic analysis of the Drp1 protein defines four regions in the molecule [21]: the head (GTPase/G domain), the neck (formed by three bundle-signalling elements (BSE) helices coming together), the middle (formed by two stalk helices coming together) and a foot formed by the variable domain (substituting the pleckstrin homology domain present in the prototype dynamin 1). In the stalk domain three interfaces have been identified regulating self-assembly and interaction with receptors anchored to the mitochondrial outer membrane: mitochondrial fission factor (MFF) and mitochondrial dynamics proteins (MID49 and MID51) [4,21,41,42].

Notably, the majority of the pathogenic variants of Drp1 fall into the stalk/bundle domain of the protein. Gly362 is part of an invariantly conserved stretch of residues forming a loop in the stalk near the foot and appears essential both for the inter-stalk interaction of Drp1 in the oligomer and in the Drp1 assembly with MID49. Indeed, substitution of Gly362 with Asp resulted in failure to co-assemble with MID49 and altered conformational properties of the oligomer [42].

Location of Ile512Thr falls in the variable domain close to the C-terminus of the first stalk segment within a stretch of residues, which is conserved in all chordates. The variable domain is an unstructured portion of Drp1 that likely undergoes conformational changes affecting self-assembly of Drp1 as well as binding to cardiolipin at the mitochondrial membranes [19,43]. The variable domain is also the site of several post-translational modifications, including sumoylation and serine-phosphorylation [44].

Our in silico modelling of the monomeric Drp1 variants 362D or 512T resulted in subtle conformational changes in the stalk/variable domain as compared with the Wt Drp1 but in a much larger conformation change in the combined 362D/512T mutant manifested by extended displacement of the 512T and distancing from the 362D. Notably, all the mutants were able to form homodimers, though with distorted geometry compared with the Wt dimer, but practically no heterodimers with the Wt monomer.

On this basis, we are tempted to suggest a pathogenic model whereby in the heterozygotic patient (362D-512T/G362-I512), the mutant Drp1, although able to form homodimers/tetramers, does not bind mitochondrial receptors nor assemble in functional oligomers around the organelle. Likely, the co-presence of the VUS I512T with the pathogenic G362D variant affects the flexibility of the variable domain that might be blocked in a conformation capping the interface site of the stalk needed for oligomer assembly [19]. This would reduce the overall ability of the cell to perform efficient mitochondrial fission (Figure 6B). The increasingly widespread genetic approach to identifying primary disease-causing variants is highlighting that no genetic variant acts alone. Other variants on the same gene or elsewhere in the genome may alleviate or exacerbate the severity of the disease, determining the variability of the phenotypic outcomes [45].

Concerning the impact of the sole VUS I512T—the condition predominantly occurring in PM—our conclusions are more tentative. Although PM did not show clinical signs of disease, her mitochondrial respiratory activity in PBMCs was as high as in P, her mitochondrial interconnected profile was intermediate between P and unrelated healthy controls, and her platelet count in the PBMCs was as low as in P. The in silico modelling of the VUS I512T homodimer resulted in altered geometry and in negligible proficiency to form heterodimers with the Wt Drp1. Noteworthy, in yeast mutant alleles of the *DNM1L*-ortholog *DNM1* carrying the two corresponding human variants (singularly and in combination), the strain carrying both variants showed a phenotype more severely affected than in that carrying the sole variant corresponding to G362D [13]. Moreover, the strain carrying the variant corresponding to I512T also showed some slight changes in the respiratory activity and in the number of petite colonies formed as compared with the Wt allele. Take together, these observations would suggest that the I512T variant is a phenotype modifier that, in combination with the G362D mutants, exacerbates its pathogenic effects. Mechanistically, we can speculate that substitution of the non-polar aliphatic Ile362 with the hydrophilic threonine in a flexible region of Dpr1 could change the intrinsic dynamics of the variable domain, influencing, though not completely hampering, the interaction of the VUS-harbouring Drp1 homodimer/homotetramer with the Wt homodimer/homotetramer, thereby resulting in a milder effect on mitochondrial fission.

## 5. Conclusions

In summary, by reassesing the case of a child affected by epileptic encephalopathy and harbouring in his *DNML1* gene the heterozygotic occurrence in cis of a pathogenic variant and of a VUS, we contributed to the spectrum of gene mutations linked to mitochondrial dynamics-related diseases and provides two further warnings. On one hand, it is called attention to the need of deepening genetic analysis by sensitive sequencing technics to eventually point out in patients’ parents low level of the gene variant harboured by the affected progeny; this would indicate hereditary transmission by a parental germline mosaicism mechanism thus contributing to improve genetic counselling. On the other hand, it is highlighted that the co-existence of pathogenic gene variants with apparently harmless variants, acting as modifiers, may influence the phenotypic outcome of the disease.

## Data Availability

Details regarding data supporting the reported results can be provided by the corresponding authors.

## Supplementary Materials

The following are available online at https://www.mdpi.com/article/10.3390/genes12091295/s1, Supplemental Material and methods; Table S1: Mitochondrial DNA sequence analysis of the patient’s PBMCs; Figure S1: Mitochondrial DNA copy number and expression of PPARGC1A in the patient’s PBMCs; Figure S2: Confocal microscopy imaging of reactive oxidant species; Figure S3: Sequence alignment of the primary structures of Drp1.
