# Supplementary material for: Pathogenic DNM1L Variant (1085G>A) Linked to Infantile Progressive Neurological Disorder: Evidence of Maternal Transmission by Germline Mosaicism and Influence of a Contemporary in cis Variant (1535T>C)
Source: Genes (Basel). 2021 Aug 24;12(9):1295. doi: 10.3390/genes12091295 (PMC8467311; doi:10.3390/genes12091295)
Supplement: Supplementary file 1 [file genes-12-01295-s001.zip › genes-1298824-supplementary.pdf]

Supplemental Materials

Supplemental Materials and Methods

Lactate assay

A lactate colorimetric assay kit (Abcam) was used following the manufacture's protocol and lactate concentration was expressed as mg/100 ml plasma.

Measurement of respiratory activity

Freshly isolated PBMCs were resuspended in 10 mM KH_2PO_4, 27 mM KCl, 1 mM $MgCl_2$, 40 mM Hepes, 0.5 mM EGTA, pH 7.1 at 4×10^6 cells/2 ml and immediately assayed for O_2 consumption by high resolution oxymetry (Oxygraph-2k, Oroboros Instruments) at 37°C under continuous stirring. After attainment of a stationary endogenous substrate-sustained respiratory rate, 2 µg/ml of oligomycin was added followed after 5 min by the addition of 1 µg/ml FCCP. The rates of oxygen consumption (OCRs) were corrected for 1 µg/ml rotenone plus 1 µg/ml antimycin A-insensitive respiration and normalized to cell number. The following mitochondrial respiratory parameters were assessed: OCR_{basal}, baseline respiration; OCR_{oligo}, H^+-leak-linked respiration measured in the presence of oligomycin; OCR_{FCCP}, maximal respiratory activity measured in the presence of the uncoupler FCCP; OCR_{ATP}, respiratory activity linked to ATP synthesis obtained subtracting OCR_{oligo} from OCR_{basal}; OCR_{spare}, reserve respiratory capacity obtained subtracting OCR_{basal} from OCR_{FCCP}.

Live Cell Imaging of ROS, Mitochondrial Membrane Potential, and Ca^{2+}.

PBMCs were seeded at low density on fibronectin-coated 35-mm glass-bottom dishes and incubated for 20 minutes at 37°C with the following probes (Molecular Probes): 2 µM tetramethylrhodamine, ethyl ester (TMRE) to monitor mitochondrial membrane potential (mtΔΨ); 10 µM 2',7'-dichlorofluorescin diacetate (DCF-DA) for detection of reactive oxidant species; 5 µM X-Rhod-1 AM for mitochondrial Ca^{2+}. Stained cells were washed with PBS and examined by a Nikon TE 2000 microscope (images collected using a 60X objective [1.4 NA]) coupled to a Radiance 2100 dual-laser confocal laser scanning microscopy system (Biorad). Acquisition, storage, and analysis of data were performed with LaserSharp and LaserPix software from Biorad and ImageJ (https://imagej.nih.gov/ij/). Morphometric analysis of the mitochondrial network was performed by ImageJ-tools with the interconnectivity index/cell calculated as mean area/mean perimeter of all the TMRE fluorescent particles thresholded within a cell.

Quantification of mtDNA copy number

Estimation of the mtDNA copy number and 28S nuclear gene were performed by quantitative PCR using the standard curve-based method[9]. The number of mtDNA copy number was normalized to cell unit (nuclear DNA was assumed to be diploid in all sample cells and the presence of 10 copies of the 28S gene for haplotype was considered).

Reverse Transcription-Polymerase Chain Reaction

For real-time reverse transcriptase (RT)-PCR, total cellular RNA was isolated using Absolutely RNA miniprep kit (Stratagene) with an on-column Dnase treatment. First-strand cDNA synthesis was carried out using 300 ng of Random Hexamers primers (Invitrogen, Carlsbad, CA, http://www.invitrogen.com) by Accuscript High Fidelity Reverse Transcriptase (Stratagene) and Ribolock Ribonuclease Inhibitor (Fermentas Life Sciences), starting from 1 µg of RNA. Real time quantification was performed with 1.5 µl of cDNA using Brilliant SYBR Green QPCR Master Mix (Stratagene) in a 25-µl reaction volume on Mx3000P (Stratagene) with 300 nM forward (for) and reverse (rev) primers (PGC1-α, for 5'-CCAAAGGATGCGCTCTCGTTCA-3', rev 5'-CGGTGTCTGTAGTGGCTTGACT-3'; β-actin, for 5'-TGGACATCCGCAAAGACCTG-3', rev 5'-GCCGATCCACACGGAGTACTT-3') and the following cycling parameters: initial denaturation for 10 minutes at 94°C; followed by cycles of 15 seconds at 94°C, 30 seconds at 60°C, and 15 seconds at 72°C; and 10 minutes of terminal elongation at 72°C. For comparative analysis the $2^{-\Delta\Delta ct}$ method was used with the β-actin transcript as internal normalizer.

Western blotting analysis

40 µg of proteins from each cell lysate, were subjected to SDS-PAGE and transferred to a polyvinylidene difluoride membrane (Bio-Rad Laboratories) using a Trans Blot Turbo Transfer System. Then, membranes were probed with the following primary antibodies against: dynamin-like protein 1 (DLP-1; 1:1000, BD Bioscience) and β-actin (1:10000, Sigma Aldrich, St. Louis, MO, USA). After incubation with a suited horseradish peroxidase-conjugated secondary antibody (1:2500; Cell Signaling Technology), signals were developed by enhanced chemiluminescence kit (Clarity Western ECL Substrate, Bio-Rad), imaged by the ChemiDoc imaging system XRS + (Bio-Rad), and analyzed using Image Lab software (version 4.1, Bio-Rad).

DNM1L gene sequencing

The variants in *DNM1L* gene (NM_012062.3) were analyzed by Polymerase Chain Reaction (PCR)-generated amplicons of DNA extracted either from PBMCs and chorionic fibroblasts[10]. Sequencing of the amplified product was performed using an automated

DNA sequencing analyzer (Applied Bio systems mod. 3130, Foster City, CA, USA), according to the manufacturer's instruction. The primers utilized were: for 5'-TGCAATGCCAGAAACCATATACT-3', rev 5'-GTAATTTCAGTTCTTCCCAAGGG-3 for the amplicon containing the c.1085G>A'; for 5'-AACTTAGTGGCAATTGAACTGG-3', rev 5'-AACAATGTAATCCCCTCAGCA-3' for the amplicon containing the c.1535T>C.

mtDNA sequencing

43 primer pairs were used to amplify the entire mitochondrial genome[11]. The overlapping PCR products were purified using the Machenery-Nagel PCR purification kit (GmbH & Co. KG) and bidirectionally sequenced using the Big Dye Terminator Cycle Sequencing Ready Reaction kit (Applied Biosystems) on an ABI Prism 3100 Genetic Analyzer (Applied Biosystems). The sequence of the patient's mtDNA was compared to the normal ‘‘Cambridge sequence’’ and deviations from it analysed using MitoAnalyzer (http://www.mitomap.org).

Structural modelling of the Wt and mutated Drp1.

The monomer structure of Wt and of Gly362Asp and/or Ile512Thr Drp1 variants was evaluated using Phyre2 software (Protein Homology-fold Recognition Server; www.sbg.bio.ic.ac.uk/phyre2/). Wild-type and mutated Drp1 3D structures (wt, 362Asp and 362Asp/512Thr) were loaded and visualized on ChemDraw (version 8; PerkinElmer). Homodimers (wt-wt, 362Asp/512Thr-362Asp/512Thr, 362Asp-362Asp and 512Thr-512Thr were generated using the GalaxyWeb tool GalaxyHomomer (http://galaxy.seoklab.org/cgi-bin/submit.cgi?type=HOMOMER) based on the pdb files of the monomeric structures obtained from Phyre2. The software generated protein homo-oligomer structures either by template-based modeling using homo-oligomer templates selected from the protein structure database or by *ab initio* docking of monomer structures resolved experimentally or predicted by computation. Heterodimers (i.e. wt-362Asp/512Thr, wt-362Asp, wt-512Thr) were generated using GRAMM-X Protein-Protein Docking Web Server v.1.2.0 (http://vakser.compbio.ku.edu/resources/gramm/grammx/). Likewise, for the production of heterodimers pdb files generated by Phyre2 were used. A total of one hundred heterodimers were processed (respectively fifty heterodimeric structures related to the possible complex of one or the other of wt-362Asp/512Thr, wt-362Asp, wt-512Thr).

Patient mtDNA variants			
A4769G	ND2	SYN-Met	known
T6776C	COX 1	SYN-His	known
A13008G	ND5	SYN-Ser	unknown
A14687G	Mt glutamyl-tRNA		known
A15326G	CYT B	Thr → Ala	known
A15758G	CYT B	Ile → Val	known
T152C	H-strand origin in D-LOOP		known
A263G	Between the two mtTF1 binding sites in D-LOOP		known
INS315C	End of the CSB2 in D-LOOP		known
T16519C	Non-coding/non-regulatory region in D-LOOP		

Table SI. **Mitochondrial DNA sequence analysis of the patient.** The indicated variants were checked against databases of previously reported benign variants (http://www.mitomap.org).

Supplemental Figures

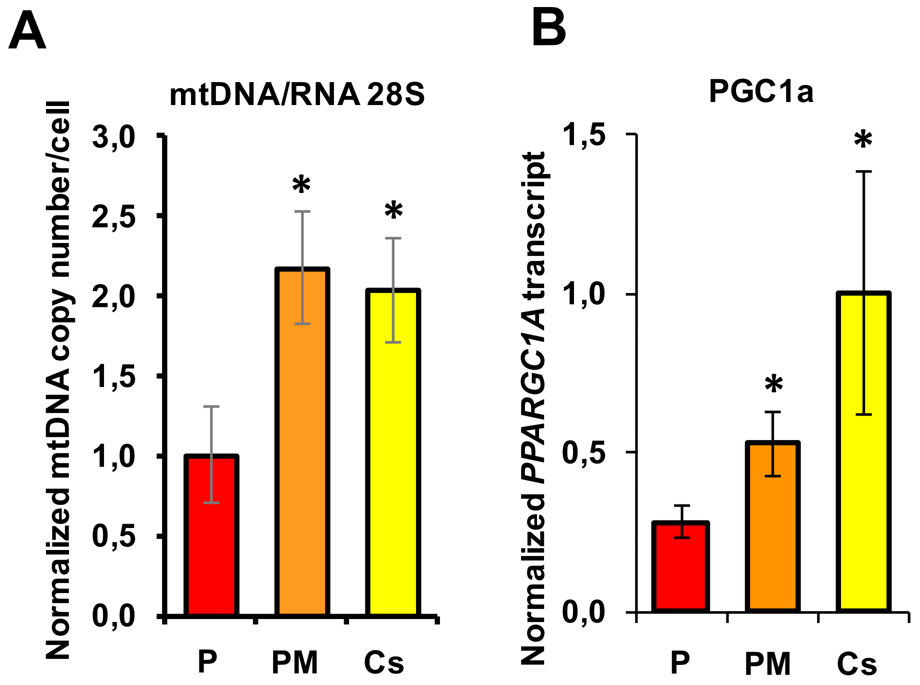

Fig. S1. (**A**) Mitochondrial DNA (mtDNA) copy number per cell using the nuclear gene RNA 28S as internal normalizer assessed by q-PCR; the values are means of three independent replicates ± SEM each carried out in triplicate and normalized to the mtDNA/cell value of P; *, P < 0.05 vs P. (**B**) Normalized transcript level of *PPARGC1A* coding for PGC1α assessed by q-RT-PCR: the values are means of three independent replicates ± SEM each carried out in triplicate; *, P < 0.05 vs P.

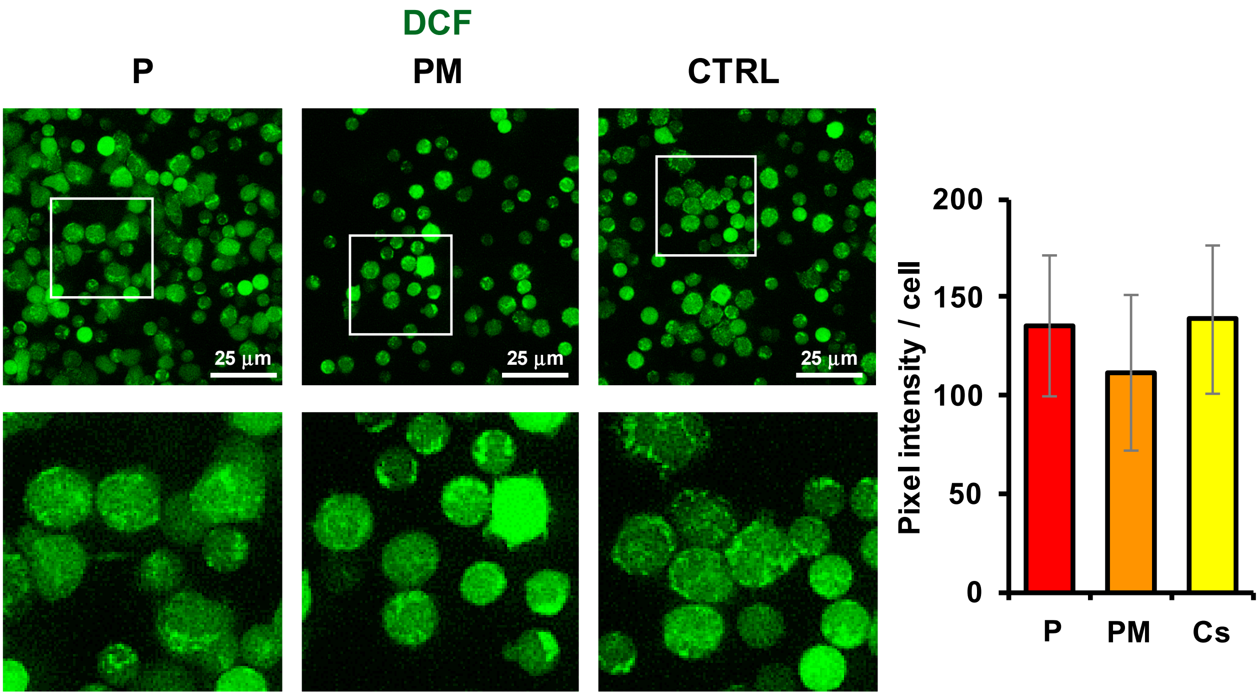

Fig. S2. Confocal microscopy imaging of reactive oxidant species. PBMCs of patient (P), patient's mother (PM) and a representative healthy subject (CTRL) were loaded with DCF-DA to assess intracellular reactive oxidant species. The tripartite picture shows representative images and digital magnifications of the squared details; the histogram on the right shows quantification of the DCF fluorescence intensity/cell; at least five different optical field/sample, each containing 40-50 cells, were examined and the values averaged ± SD from three independent biological preparations.

```
Drp1                             362                                      512
                                  ⇓                                        ⇓
H.sapiens       AKYIETSELCGGARICYIFHETFGRTLE     DFADA---CGLMNNNIEEQRR-------
P.troglodytes   AKYIETSELCGGARICYIFHETFGRTLE     DFADA---CGLMNNNIEEQRR-------
M.mulatta       AKYIETSELCGGARICYIFHETFGRTLE     DFADA---CGLMNNNIEEQRR-------
C.Lupus         AKYIETSELCGGARICYIFHETFGRTLE     DFADA---CGLMNNNIEEQRR-------
B.taurus        AKYIETSELCGGARICYIFHETFGRTLE     DFADA---CGLMNNNIEEQRR-------
M.musculus      AKYIETSELCGGARICYIFHETFGRTLE     DFADA---CGLMNNNIEEQRR-------
R.norvegicus    AKYIETSELCGGARICYIFHETFGRTLE     DFADA---CGLMNNNIEEQRR-------
G.gallus        AKYIETSELCGGARICYIFHETFGRTLE     DFADA---CGLMNNNIEEQRR-------
X.tropicalis    AKYIETSELCGGARICYIFHETFGRTLE     DFADA---CGLMNNNIEEQRR-------
D.rerio         AKYIETAELCGGARICYIFHETFGRTLE     DFADA---CGLMNNNIEEQRR-------
D.melanogaster  ARNIETTELCGGARMGYIFHETFGRTLD     DFHKD---AALVPSLLKTDSDPYSQINL
A.gambiae       SRNIETTELCGGARICYIFHETFGKTLD     DFHKD---AALVPSLIKTDSQ-------
C.elegans       ARNIETTELCGGARICYIFHDTFGRSLE     EFTEANLVTLLKEELLLDDRH-------
S.cerevisiae    SSDINTKELCGGARIYYIYNNVFGNSLK     NFLSA---TEAMDDIMKTRRKRNQ----
K.lactis        SSEISTKELCGGARIYYIYNTLFGKSLN     NFPSA---TEAMAEIVEARKNKKQTERL
E.gossypli      SSDISTKELCGGARIYYIYNNIFGNSLK     NFLSA---TEAMADIAEARRQKQQNAKR
S.pompe         SSNIPTKELSGGARLYSIFNNVFTTALN     DFLGV---QGAMAVVLS--RKEQNRLML
M.oryzae        STEISTKELCGGARIYYIFNSVFGSSLE     NFLGA---TAAMSNVVSEKQERERKKII
N.crassa        STEISTKELCGGARIYYIFNSVFGSSLE     NFLGA---AAAMSHVVSNKQERERKRLI
O.sativa        NK-VSTDELSGGARIHYIFQSIFVKSLE     NFVGG-------NKVVELARQ-------
```

Fig. S3. Sequence alignment of the primary structures of Drp1. The sequence numbering of the indicated residues G362 and I512 (highlighted in light-blue) refers to the human Drp1; invariant residues are highlighted in yellow.
